# Quercetin and Cardiovascular Health: An Evidence‐Based Review of Risk Factors and Treatments

**DOI:** 10.1002/fsn3.72514

**Published:** 2026-10-28

**Authors:** Mohammad Reza Khazdair, Shima Jafari, Melika Shoghi

**Affiliations:** ^1^ Cardiovascular Diseases Research Center Birjand University of Medical Sciences Birjand Iran; ^2^ Department of Clinical Pharmacy, School of Pharmacy Birjand University of Medical Sciences Birjand Iran; ^3^ Student Research Committee Birjand University of Medical Sciences Birjand Iran

**Keywords:** hypertension, hypertrophy, ischemia, lipid profile, quercetin, risk factors

## Abstract

Cardiovascular diseases (CVD) encompass a variety of conditions, including coronary heart disease, peripheral arterial disease, cerebrovascular disease, rheumatic heart disease, and venous thromboembolism. Natural products exhibit a range of pharmacological activities, including antioxidant, anti‐inflammatory, and immunoregulatory effects. This review evaluates the potential therapeutic effects of quercetin on factors contributing to cardiovascular disease risk. Quercetin, a flavonoid recognized for its strong antioxidant capabilities, has demonstrated beneficial effects in alleviating pathological cardiac conditions such as hypertrophy and ischemia across multiple in vitro and in vivo experimental models. This review compiles evidence demonstrating quercetin's ability to reduce cardiac hypertrophy and apoptosis in cardiomyocytes through modulation of key signaling pathways. Specifically, quercetin treatment resulted in significant upregulation of protective proteins such as silent information regulatory factor 1 (SIRT1), peroxisome proliferator‐activated receptor‐γ coactivator‐1α (PGC‐1α), and Bcl‐2, coupled with downregulation of pro‐apoptotic Bax. Quercetin improved calcium channel function, enhanced myocardial contractility, and decreased oxidative stress markers, leading to improved survival rates of cardiomyocytes under ischemic conditions. Quercetin administration has been shown to lower systolic and diastolic blood pressure and reduce cardiac hypertrophy. Additionally, quercetin positively influences lipid profiles by decreasing total cholesterol, triglycerides, and low‐density lipoprotein while enhancing overall lipid metabolism. Collectively, these findings highlight quercetin as a promising candidate in preclinical models of cardiac hypertrophy and ischemia. However, further research—particularly well‐designed clinical trials—is essential to validate these findings and determine the translational potential through dietary interventions of quercetin in human cardiovascular health.

## Graphical abstract

Quercetin is a naturally occurring polyphenolic phytochemical distributed in fruits and vegetables. Quercetin, as a flavonoid, showed significant antioxidant capabilities and potential therapeutic effects on CVD risk factors. Quercetin reduces cardiac hypertrophy and apoptosis in cardiomyocytes by modulating critical signaling pathways. Quercetin enhances calcium channel function, improves myocardial contractility. Quercetin shows inhibitory effects on inflammatory pathways (Src kinase, STAT3), suggesting its role in reducing cardiac stress and inflammation.

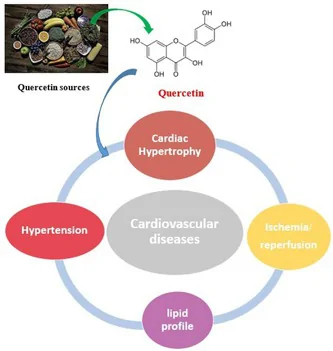

## 1 Introduction

Cardiovascular diseases (CVD) involve disorders of the circulatory system, specifically affecting the heart and blood vessels (Biglu et al. 2016). According to the World Health Organization (WHO), CVDs remain the leading cause of global mortality, accounting for approximately 31% of all deaths worldwide (Benjamin et al. 2017). These conditions are classified into several types, including coronary heart disease, peripheral arterial disease, cerebrovascular disease, rheumatic heart disease, and congenital heart disease (Alotaibi et al. 2021). The WHO suggests that up to 75% of CVD cases may be prevented by addressing modifiable risk factors, such as diabetes, dyslipidemia, hypertension, obesity, physical inactivity, and tobacco use (Mahmood et al. 2014).

In addition to lifestyle modifications, pharmacotherapies including angiotensin‐converting enzyme (ACE) inhibitors, calcium channel blockers, anticoagulants, antiplatelet agents, vasodilators, lipid‐lowering drugs, and 𝛽‐blockers are routinely employed for the primary prevention and management of cardiovascular conditions (Li et al. 2015).

In recent decades, there has been a significant increase in the use of medicinal plants for health promotion and disease management, even in developed economies. Numerous botanical extracts are now utilized as formal prescription medications in countries such as the United Kingdom, Germany, China, and France (Yatoo et al. 2017). Clinical assessments generally indicate that herbal medicines are better tolerated than many synthetic pharmaceuticals (Izzo et al. 2016; Khazdair et al. 2020).

Furthermore, approximately one‐third of FDA‐approved drugs developed over the past two decades are derived from natural products or their derivatives (Patridge et al. 2016). The structural diversity and specificity of plant‐derived compounds make them a valuable resource for new drug discovery in cardiovascular medicine (Alotaibi et al. 2021). *For instance, phenolic acids and polyphenols possess diverse biological* properties. Gallic acid, a phenolic compound, has demonstrated therapeutic efficacy in CVDs, including protection against drug‐induced cardiotoxicity, hypertension, myocardial ischemia–reperfusion injury, and infarction (Momeni et al. 2024). Similarly, the role of the polyphenol epigallocatechin gallate has been investigated for its potential in preventing hypertension and atherosclerosis (Eshraghi et al. 2025).

Flavonoids are a broad superfamily of phytochemicals characterized by potent antioxidant properties and are categorized into classes including flavonols, flavones, flavanones, anthocyanidins, and isoflavones (Falcone Ferreyra et al. 2012; Winkel‐Shirley 2001). A prominent flavonol subclass, polyphenol, is primarily sourced from plants such as *Portulaca oleracea* and *Allium cepa* (Khazdair et al. 2019, 2021). Quercetin, one of the most abundant dietary flavonoids, is a naturally occurring polyphenolic phytochemical distributed in fruits, vegetables, and various medicinal plants. Its principal daily dietary sources include onions, apples, and tea (Vollmannová et al. 2024).

Accumulating evidence highlights the multifaceted pharmacological profile of quercetin, encompassing anti‐inflammatory, vasodilatory, anti‐carcinogenic, anti‐proliferative, and antioxidant properties, which render it a promising therapeutic agent across diverse pathological conditions (David et al. 2016; Sultana and Anwar 2008). In clinical settings, quercetin has been shown to reduce blood pressure in hypertensive individuals and to lower the associated risk of cardiovascular disease. Additionally, it appears to alleviate post‐exercise inflammation and muscle damage (Yan et al. 2023).

Given that numerous studies have linked ubiquitous dietary occurrence, potent antioxidant and anti‐inflammatory properties, and favorable safety profile, it is imperative to critically evaluate the existing evidence regarding its multifaceted impact on cardiovascular risk factors.

## 2 Effects of Quercetin on Cardiac Hypertrophy

### 2.1 In Vitro Studies

The reagent quercetin glucuronide (1–100 μM) demonstrated a concentration‐dependent inhibition of Angiotensin II (Ang II)‐induced Jun N‐terminal kinase (JNK) activation, while it had no effect on the activation of extracellular signal‐regulated kinase 1/2 (ERK1/2) or p38 MAP kinase. Quercetin exhibited radical‐scavenging properties against DPPH radicals in rat aortic smooth muscle cells (RASMC). Additionally, quercetin inhibited Ang II‐induced increases in activator protein 1 (AP‐1) DNA binding activity in a concentration‐dependent manner, mirroring its suppression of JNK activation. These findings represent a novel contribution, showing that quercetin glucuronide, a circulating metabolite of quercetin, impedes Ang II‐induced hypertrophy in RASMC through inhibition of both JNK and the AP‐1 signaling pathway (Yoshizumi et al. 2002).

Further analyses revealed that quercetin (4 μg/mL) enhanced superoxide dismutase (SOD) activity and decreased total protein content, the size of cardiomyocytes, MDA production, and expressions of ERK1/2, JNK, and p38, thereby attenuating hypertrophy and JNK activation in rat cardiomyocytes induced by Ang II (Wu and Gu 2006). Similarly, in primary cultures of neonatal rat cardiomyocytes, quercetin (Que) enhanced SOD activity and diminished MDA production in response to Ang II‐induced hypertrophy. Quercetin also significantly inhibited the activation of ERK1/2, JNK, and p38 upon Ang II stimulation and reduced Ang II‐induced early response gene expression of c‐fos mRNA (Wu and Gu 2007). In another in vitro study, quercetin (100 μg/mL) exposure significantly decreased Ang II‐induced hypertrophy in H9C2 cardiomyocytes, inhibiting the mRNA expression of hypertrophic biomarkers such as atrial natriuretic peptide (ANP) and brain natriuretic peptide (BNP) in these cells (Yan et al. 2013).

The impact of quercetin on oxidative stress and the mitogen‐activated protein kinase (MAPK) pathway in H9C2 cardiomyocytes subjected to Ang II‐induced hypertrophy indicated that quercetin (331 μM) significantly downregulated the phosphorylated forms of ERK1/2, p38, and JNK1/2 (Siti et al. 2021). Furthermore, while Ang II elevated mRNA levels of hypertrophic markers like atrial natriuretic factor (ANF) and β‐myosin heavy chain (β‐MHC) in H9C2 cells, quercetin (2 μM) effectively mitigated this hypertrophic response and preserved mitochondrial function against Ang II exposure (Chen et al. 2021).

Quercetin, particularly in its glucuronide form Q3GA, significantly alleviates Ang II‐induced cardiac hypertrophy and oxidative stress across various cellular models. The capacity of quercetin to inhibit JNK activation and the AP‐1 signaling pathway underscores a specific mechanistic approach through which it exerts its protective effects on RASMC and cardiomyocytes. The influence of quercetin on cardiac hypertrophy in in vitro studies is summarized in Figure 1.

**FIGURE 1:**
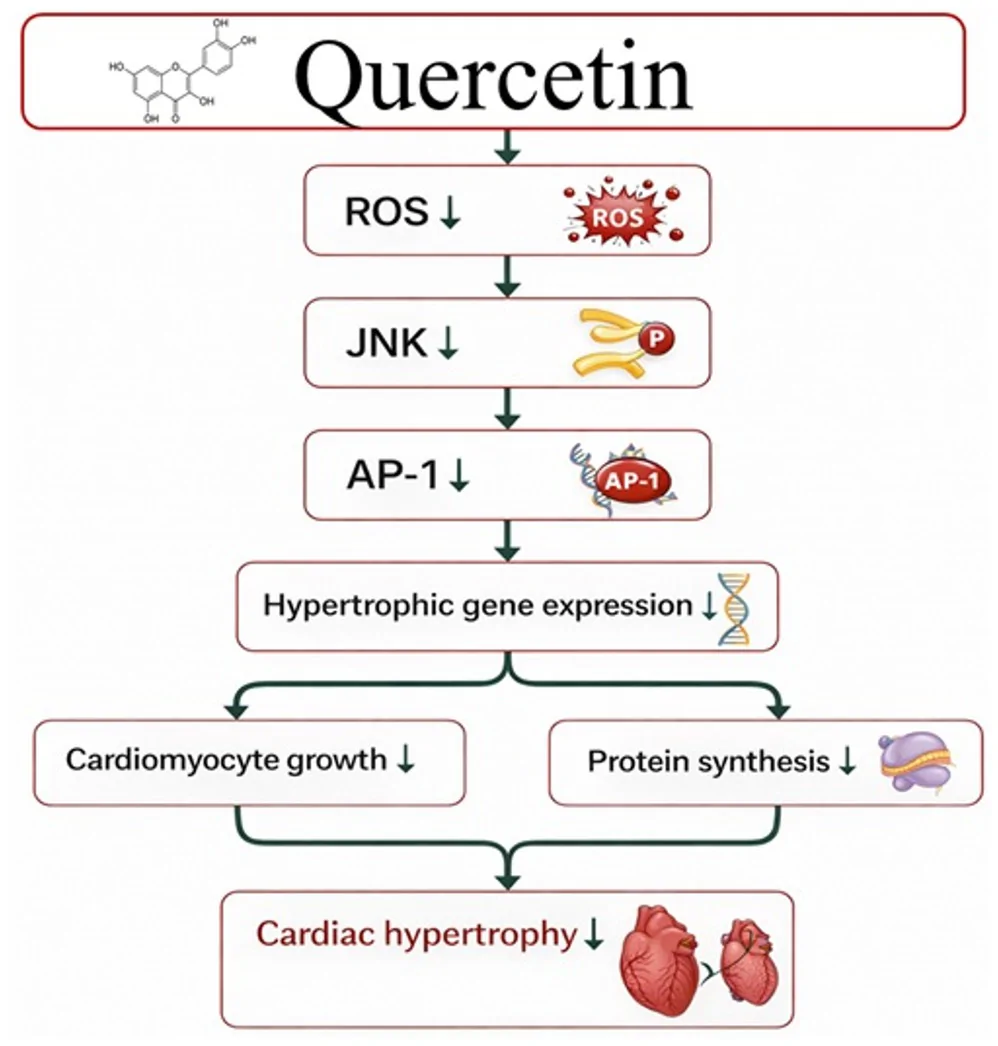
Effects of quercetin on cardiac hypertrophy in in vitro studies.

### 2.2 In Vivo Studies

Research investigating the effects of quercetin on pathological cardiac hypertrophy has utilized mouse models and revealed significant increases in antioxidant activity. Administration of quercetin at a dosage of 10 mg/kg/day effectively blocked hydrogen peroxide (H_2_O_2_), enhanced mitochondrial SOD activity, and restored protein sulfhydryl groups, thereby protecting mitochondrial ion channels and alleviating mitochondrial permeability during cardiac hypertrophy transitions. Furthermore, quercetin reversed cardiac hypertrophy induced by isoproterenol by restoring mitochondrial protection and re‐establishing cellular redox balance (de Lacerda Alexandre et al. 2021). In a study involving rats and primary neonatal cardiomyocytes, quercetin significantly improved cardiac diastolic function and glycogen synthase kinase 3 (GSK‐3) activity while inhibiting proteasome activity. Notably, it activated GSK‐3α/β and concurrently reduced cardiac hypertrophy, Angiotensin II (Ang II)‐induced increases in hypertrophic surface area. Quercetin administration also decreased atrial natriuretic factor (ANF) mRNA levels and proteasome activity, including the subunit levels of β‐type proteasome subunits (PSMB)1, PSMB2, PSMB5, and regulatory particles (Rpt1 and Rpt4). Quercetin also decreased the levels of phosphorylated GSK‐3α/β, indicating GSK‐3 inactivation, and reduced the phosphorylation of histone H3, β‐catenin, and GATA binding protein 4 (GATA4) (Chen et al. 2018). Treatment of induced cardiac hypertrophy rats by blocking AP‐1 (c‐fos, c‐jun) with quercetin (5–10 mg/kg) via gavage significantly increased PPAR‐γ protein and PPAR‐γ expression, activating PPAR‐γ signaling pathways, as shown by its effects on pathological heart hypertrophy. It also reduced blood pressure, the ratio of LV to body weight, cardiac hypertrophy, downstream hypertrophy gene, mRNA levels of atrial natriuretic peptide (ANP), and B‐type natriuretic peptide (BNP), AP‐1 protein in rats (Yan et al. 2013). In rats with pathological cardiac hypertrophy, oral administration of quercetin at a dose of 1.5 g/kg decreased blood pressure, carotid arterial blood pressure, hepatic lipid oxidation, cardiac hypertrophy, expression of cardiac beta‐myosin heavy‐chain mRNA, and aortic thickening. However, quercetin increased the antihypertensive and anti‐hypertrophic effects instead of all of these factors (Jalili et al. 2006).

In spontaneously hypertensive rats (SHRs), intragastric administration of quercetin (20 mg/kg/day) improved cardiac function, reduced mitochondrial superoxide levels, and preserved mitochondrial structure, while also decreasing cardiac hypertrophy and superoxide production. Additionally, in vitro studies indicated that Ang II increased mRNA levels of hypertrophic markers such as ANF and β‐myosin heavy chain (β‐MHC), yet quercetin mitigated this hypertrophic response and safeguarded mitochondrial function against Ang II induction (Chen et al. 2021). Furthermore, daily administration of quercetin (5 or 10 mg) in drinking water over 3 weeks markedly reduced cardiac hypertrophy, as reflected by the heart weight‐to‐body weight (HW/BW) ratio, and decreased levels of MDA, a product of lipid peroxidation, and signal‐regulated kinase (ERK1/2). Quercetin also diminished protein expression and phosphorylation activity of Akt and GSK‐3β, as well as P38 MAP kinase and JNK1/2 in hearts subjected to pressure overload in a rat model of pathological cardiac hypertrophy (Han et al. 2009). In a mouse model of pathological cardiac hypertrophy, low‐dose oral quercetin (0.1 μmol) given over 6 weeks significantly reduced total cholesterol and very low‐density lipoprotein levels in the plasma of ApoE mice. Additionally, quercetin treatment resulted in significant reductions in left ventricular (LV) posterior wall thickness and LV mass in ApoE mice (Ulasova et al. 2013). Collectively, these findings indicate that quercetin exerts notable protective effects against cardiac hypertrophy in diverse animal models, primarily through enhanced antioxidant activity, improved mitochondrial function, and modulation of critical signaling pathways associated with cardiac hypertrophy. Quercetin or quercetin glucuronide enhances antioxidant enzyme activities, specifically SOD, catalase, and GPx, while reducing MDA and H_2_O_2_ levels. In mitochondrial protection, by blocking H_2_O_2_ and restoring protein sulfhydryl groups, quercetin prevents the loss of mitochondrial membrane potential and protects mitochondrial ion channels.

The transition from oxidative stress to cellular hypertrophy is mediated by several key signaling including: (1) JNK and AP‐1, Quercetin shows high specificity for the JNK/AP‐1 pathway. By inhibiting JNK activation, quercetin prevents the subsequent binding of the AP‐1 transcription factor (c‐fos, c‐jun) to DNA, thereby blocking the transcription of early‐response hypertrophic genes. (2) ERK1/2 and p38, some studies show varying effects on ERK1/2 and p38, the consensus indicates that quercetin limits the phosphorylation (activation) of these kinases, effectively “de‐tuning” the cell's response to Ang II stimulation. The data are now presented as a defined cascade:

Quercetin → ↓ROS/↑Nrf2–ARE transcription → ↓oxidative stress → restored mitochondrial biogenesis and function (↑PGC‐1α, ↑TFAM, ↑ATP, ↑complex activity) → normalized intracellular lipogenic flux (↑AMPK → ↓ACC, ↓SREBP‐1c, ↓FASN) → reduced lipid accumulation/lipotoxicity (↓blood lipids) → attenuated MAPK signaling (↓p‐ERK1/2, ↓p‐p38, ↓p‐JNK) and NFATc3 activation → suppression of cardiac hypertrophy (↓cardiomyocyte size, ↓hypertrophic gene expression). The effects of quercetin on cardiac hypertrophy in in vitro studies are summarize in Table 1 and describe in Figure 2.

**TABLE 1:** Effects of quercetin on cardiac hypertrophy.

Study design	Doses	Route of administration	Effects	References
Rat aortic smooth muscle cells (RASMC)	1–100 μM	Expose	↑Radical‐scavenging activity ↓AP‐1 DNA binding activity ↓Ang II‐induced RASMC hypertrophy ↓JNK and the AP‐1 signaling pathway	Yoshizumi et al. (2002)
Rat cardiomyocyte	4 μg/mL	Exposed	↓Total protein content ↓Cell size of cardiomyocytes ↑SOD activity ↓Production of MDA ↓ERK1/2, JNK and P38 expressions ↓Hypertrophy ↓JNK activation induced by AngII	Wu and Gu (2006)
Rat cardiomyocyte	1–100 μmol/L	Exposed	↓Total protein content ↓Cell size of cardiomyocytes ↑SOD activity ↓Production of MDA ↓JNK activation ↓Early response gent c‐fos mnRNA expression ↓Cardiomyocyte hypertrophy	Wu and Gu (2007)
H9C2 cells	100 μg/mL	Exposed	↓Ang II‐induced H9C2 cells hypertrophy ↓mRNA expression levels of genes such as ANP and BNP in hypertrophic H9C2 cells	Yan et al. (2013)
Cardiomyocyte	331 μm	Exposed	↓Phosphorylated proteins of ERK1/2, p38, and JNK1/2. ↓JNK1/2	Siti et al. (2021)
Rat cardiomyocyte	1–100 μmol/L	Exposed	↓Total protein content ↓Cardiomyocytes hypertrophy ↓Effect on PKC and TPK	Qin et al. (2001)
Mice	10 mg/kg/day	Osmotic pumps	↓Cardiac hypertrophy ↑Mitochondrial SOD activity ↓Cardiac hypertrophy ↑Cellular redox balance and protecting mitochondria Restore protein sulfhydryl ↑Antioxidant activity	de Lacerda Alexandre et al. (2021)
Rats	—	—	↓Cardiac hypertrophy ↑Cardiac diastolic function ↓Ang II‐induced hypertrophic surface area ↓Atrial natriuretic factor (ANF) mRNA level ↑GSK‐3 activity ↓Phosphorylation levels of upstream AKT (PKB (protein kinase B)) ↓Liver kinase B1/AMP ↓Signal‐regulated kinase (ERK) ↓Histone H3, β‐catenin ↓GATA binding protein 4 (GATA4) ↓Cardiac hypertrophy ↑Proteasome inhibition	Chen et al. (2018)
Rats	5 mg/kg 10 mg/kg	Gavage	↓Cardiac hypertrophy ↑Activating PPAR‐γ signaling pathways. ↓Ratio of left ventricular to body weight ↓AP‐1 (c‐fos, s‐jun) ↓Downstream hypertrophy gene ↓MRNA levels of ANP and BNP ↑PPAR‐γ protein ↓AP‐1 protein ↓MRNA expression ↓Cardiac hypertrophy ↑PPAR‐γ expression ↓AP‐1 signaling pathway	Yan et al. (2013)
Rats	1.5 g/kg	Chow	↓Blood pressure ↓Signal transduction pathways ↓Hepatic lipid oxidation ↓Carotid arterial blood pressure ↓Cardiac hypertrophy ↓Expression of cardiac beta‐myosin heavy‐chain mRNA ↓Aortic thickening	Jalili et al. (2006)
Rats	20 mg/kg/day	Intragastrical	↑Cardiac function ↓Mitochondrial superoxide ↑ Mitochondrial function ↓Cardiac hypertrophy	Chen et al. (2021)
Rats			↓Cardiac hypertrophy ↓Malonyldialdehyde production ↓Signal‐regulated kinase (ERK1/2), ↓P38 MAP kinase ↓Akt and GSK‐3beta ↓Oxidant status ↓ERK1/2, p38 MAP kinase ↓Akt and GSK‐3beta activities	Han et al. (2009)
Rats	1 g	Oral	↓Angiotensin II ↓C‐Jun N‐terminal kinase (JNK) activation ↓JNK activation ↓Activator protein‐1 (AP‐1) DNA binding ↓[3H]leucine incorporation ↓VSMC hypertrophy ↓Effect on JNK ↓AP‐1 signaling pathway	Wu and Gu (2006)
Mice	0.1 μmol QCN/kg	Oral	↓Total cholesterol ↓Very low‐density lipoprotein ↓LV posterior wall thickness ↓LV mass in Apo E (−/−) mice ↓Maladaptive myocardial remodeling	Ulasova et al. (2013)
Wistar rats	10 μmol NaQSA or 50 μmol NaQSA	Extracorporeal liver perfusion	↑DDAH activity ↓ADMA concentration	Trocha et al. (2012)
Mice	3.5 μg/mL 2.36 μg/mL	Intestinal perfusion	↑Phase II metabolites in perfusion ↑Plasma and bile samples	Orrego‐Lagarón et al. (2016)
Rats	25–300 μmol 50–300 μmol	Perfusion	↓NADH to NAD (+) ratio ↑Stimulated the Krebs cycle ↑NAD (+) ↓NADH availability ↓Mitochondrial energy ↓Oxygen uptake	Buss et al. (2005)

**FIGURE 2:**
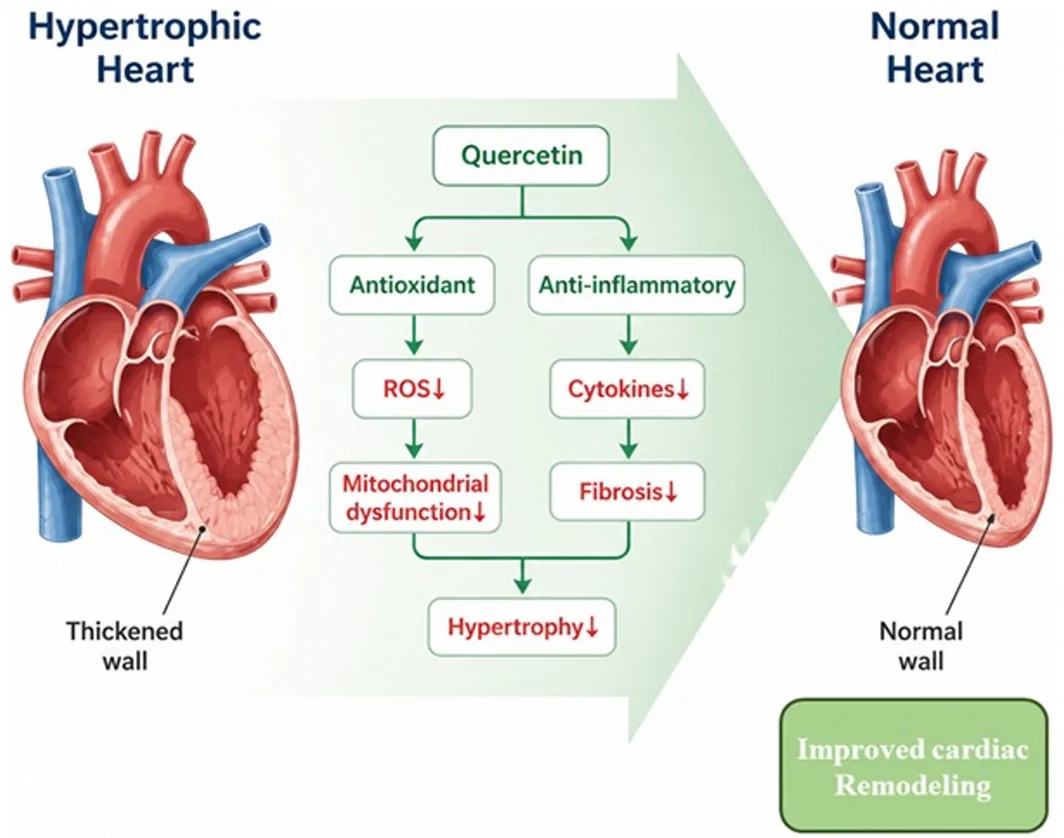
Effects of quercetin on cardiac hypertrophy in in vivo studies.

## 3 Effects of Quercetin on Ischemia/Reperfusion

### 3.1 In Vitro Studies

Investigations into the effects of quercetin on pathological cardiac ischemia in cardiomyocytes revealed that exposure to quercetin at concentrations ranging from 25 to 100 mL/L significantly reduced apoptosis rates compared to the control group. Quercetin treatment led to a substantial upregulation of the silent information regulatory factor 1 (SIRT1), peroxisome proliferator‐activated receptor‐γ coactivator‐1α (PGC‐1α), and Bcl‐2 protein expressions, while the expression of the pro‐apoptotic protein Bax was notably downregulated (Tang et al. 2019). Moreover, quercetin demonstrated a half‐maximal inhibitory concentration of 4.67 × 10^−4^ M, which positively influenced Ca^2+^ channel activity in cardiomyocytes subjected to ischemic conditions. The treatment improved cellular mechanisms related to L‐type Ca^2+^ channels (LTCC), shifting the activation and inactivation curves of the current–voltage relationship to the left. This inhibition of LTCC translated into cardioprotective effects, enhancing myocardial contractility while decreasing J‐point elevation, heart rate, levels of reactive oxygen species (ROS), serum myocardial enzyme levels, and enhancing superoxide dismutase, catalase, glutathione, glutathione peroxidase, and glutathione S‐transferase activities. It also positively influenced the amplitude of cell shortening, Ca^2+^ transients, and Ca^2+^ influx in rats (Liang et al. 2020).

Additionally, quercetin administration (1 mM) in cardiomyocytes subjected to pathological cardiac ischemia resulted in a reduction in Src kinase activity, signal transducer and activator of transcription 3 (STAT3) activity. The intracellular ROS production, protein metabolism, caspase‐9 activity, and expression levels of inflammatory markers, as well as inducible manganese superoxide dismutase (MnSOD), Bax, and lipid metabolism‐related factors were reduced after treatment (Chen et al. 2013). Overall, quercetin exhibited significant cardioprotective effects against ischemic conditions in cardiomyocytes through its ability to reduce cell apoptosis and modulate key apoptotic and survival proteins, including SIRT1, PGC‐1α, Bcl‐2, and Bax. Furthermore, quercetin enhanced calcium channel activity, thereby improving myocardial contractility while simultaneously decreasing markers of oxidative stress and mitigating inflammatory responses.

### 3.2 In Vivo Studies

Administration of quercetin significantly ameliorated pathological myocardial morphology and markedly reduced the rates of cell apoptosis compared to the control group. In a rat model, treatment with quercetin (25–100 mg/kg) significantly upregulated the protein expressions of silent information regulator 1 (SIRT1), peroxisome proliferator‐activated receptor‐γ coactivator‐1α (PGC‐1α), and Bcl‐2. Moreover, quercetin downregulated Bax expression, indicating that quercetin protects against cardiomyocyte apoptosis induced by myocardial ischemia/reperfusion through the SIRT1/PGC‐1α signaling pathway (Tang et al. 2019). In a study involving a rat model of cardiac ischemia, quercetin administration (10–40 μM) significantly enhanced the Janus kinase 2/signal transducer and activator of transcription 3 (JAK2/STAT3) pathway, improved cardiac function‐related biochemical indices, and reduced cell apoptosis, oxidative stress, and extents of myocardial infarction (Liu, Yao, et al. 2021).

In another model focusing on myocardial ischemia–reperfusion injury in diabetic fatty rats, daily administration of quercetin at 20 mg/kg for 6 weeks resulted in increased vascular relaxation and upregulated expression of endothelial nitric oxide synthase (eNOS) and protein kinase C epsilon (PKCϵ) (Ferenczyova et al. 2020). Furthermore, intravenous injection of quercetin (1 mg/kg) in a rabbit model significantly reduced the expression of NADPH oxidase 2 (Nox2), eNOS, and inducible nitric oxide synthase (iNOS) at both mRNA and protein levels following myocardial ischemia–reperfusion injury (MIRI) (Wan et al. 2009). Similar findings were observed in a rat model, where quercetin administration (1 mg/kg) led to significant anti‐inflammatory effects by reducing tumor necrosis factor‐alpha (TNF‐α), decreasing infarct volume, and improving hemodynamic abnormalities (Jin et al. 2012). Additional intravenous administration of quercetin (100 nM) resulted in significantly enhanced left ventricular developed pressure (LVDP) and reduced levels of creatine kinase (CK), while also diminishing inflammatory markers such as interleukin‐1 beta (IL‐1β), IL‐6, and TNF‐α. Moreover, it positively influenced mitochondrial K‐ATP (mitoKATP) channels and nitric oxide (NO) signaling, implicating these pathways in quercetin's anti‐inflammatory effects (Liu, Yao, et al. 2021). These results indicated that the anti‐inflammatory effects of quercetin might be involved through mitoKATP channels and NO system. In a rat model receiving daily quercetin administration (20 mg/kg/day) for 6 weeks, there was marked improvement in vascular relaxation and increased expression of eNOS and PKCϵ, alongside a reduction in systolic blood pressure (Ferenczyova et al. 2020). Additionally, intravenous quercetin (1 mg/kg) decreased NOX2 and iNOS expression levels, illustrating its efficacy in the context of induced cardiac ischemia in rabbits (Wan et al. 2009).

Intravenous administration of quercetin (1.0 mg/kg) significantly increased anti‐inflammatory effects, decreased infarct volume, and improved the baseline hemodynamic abnormalities. Quercetin also reduced TNF‐α and IL‐10 levels in MIRI rats (Jin et al. 2012). Oral administration of quercetin at a dose of 250 mg/kg resulted in enhanced cardiac function, increased Bcl‐2 levels, and Akt phosphorylation, illustrating its potential for anti‐apoptotic effect alongside reductions in apoptosis rates. It also decreased markers of myocardial injury such as CK, lactate dehydrogenase (LDH), and aspartate aminotransferase (AST). Furthermore, quercetin exhibited antioxidant and anti‐inflammatory properties by reducing malondialdehyde (MDA) levels while increasing glutathione (GSH) and activities of superoxide dismutase (SOD), catalase (CAT), glutathione peroxidase (GSH‐Px), and glutathione reductase (GR) (Liu et al. 2014). Quercetin administration (1 mg/kg) during reperfusion led to significant improvements in heart function, coronary flow, myocardial contractility, and activation of the high mobility group box 1 (HMGB1) pathway while decreasing myocardial injury and inflammatory cytokine production in a model of coronary artery occlusion‐induced ischemia in rats (Dong et al. 2018). Subcutaneous administration of quercetin (20 mg/kg) significantly reduced MDA levels, pro‐apoptotic marker caspase‐3, contractile responses to carbachol, and myeloperoxidase (MPO) activities. Quercetin also increased bladder tissue contractility against acute ischemia/reperfusion (I/R) injury, GSH levels, SOD activities, and anti‐apoptotic bcl‐2 protein compared to the control group (Tinay et al. 2017). In murine models, intraperitoneal administration of quercetin (10 mg/kg/day) notably reduced infarct size and serum levels of creatine kinase and lactate dehydrogenase, along with diminished cardiomyocyte apoptosis and caspase‐3 activation, downregulating Bax expression and increasing phosphorylated Akt and Bcl‐2 levels (Wang et al. 2013). Moreover, oral gavage of quercetin at 250 mg/kg/day enhanced PPARγ activation, improved cardiac function, and mitigated myocardial damage, infarct size, oxidative stress, apoptosis, and suppressed the activation of the NF‐κB pathway following myocardial ischemia–reperfusion injury (Liu et al. 2016).

Administration of quercetin at a concentration of 15 μM/L significantly facilitated post‐ischemic recovery after reperfusion, improving LVDP, and increasing maximal rates of pressure development (+dP/dt(max)) and decline (‐dP/dt(max)), while also significantly reducing infarct size (Bartekova et al. 2010). Daily quercetin glucosides (100 mg/kg/day) in a model of induced hindlimb ischemia in older male mice significantly enhanced recovery of blood flow to the ischemic leg with an observed increase in capillary density in the ischemic muscle without promoting tumor growth (Sumi et al. 2013). Overall, quercetin appears to significantly enhance antioxidant properties, reduce infarct size, serum markers of myocardial injury, and apoptosis, while improving cardiac function and promoting beneficial cellular pathways, such as PPARγ activation and Akt phosphorylation, across various experimental models.

### 3.3 Clinical Studies

In a randomized clinical trial (RCT), the administration of quercetin (120 mg/day) as an adjunct to standard treatment in patients with stable coronary heart disease (CHD) resulted in significant improvements in left ventricular systolic and diastolic functions, as well as deceleration time (DT). Furthermore, quercetin administration led to a reduction in the total number of ST‐segment depression episodes and premature ventricular contractions (PVCs) (Chekalina et al. 2017).

The cardioprotective effect of quercetin on limiting infarct size in patients with ST‐segment elevation myocardial infarction (STEMI) was also investigated. In this study, intravenous infusion of quercetin (500 mg) was initiated prior to reperfusion and repeated over the subsequent 5 days. The median early creatine kinase‐myocardial band area under the curve (CK‐MB AUC) was significantly lower in the quercetin group compared to the control group. Additionally, quercetin administration was significantly associated with a reduction in reperfusion‐induced intramyocardial hemorrhage, as assessed by cardiac magnetic resonance imaging on Day 3 (11.1% vs. 53.3% in the control group) (Kozhukhov et al. 2024). The efficacy of quercetin in reducing myocardial injury and senescence in patients undergoing coronary artery bypass grafting (CABG) following acute coronary syndrome was evaluated in a randomized, double‐blind clinical trial (RCT). Participants received either quercetin supplementation (500 mg twice daily) or a placebo, starting 2 days preoperatively and continuing until the seventh postoperative day. The results indicated that quercetin supplementation limited the vigorous inflammatory response triggered by CABG and may also improve endothelial function by eliminating senescent vascular endothelial cells (Dagher et al. 2021). Furthermore, an RCT conducted on 88 post‐myocardial infarction (MI) patients demonstrated that receiving quercetin supplementation (500 mg/day) for 8 weeks significantly reduced TNF‐α levels, increased serum total antioxidant capacity, and improved the insecurity dimension of the quality of life compared to the placebo group (Dehghani et al. 2021). The clinical evidence indicated that quercetin demonstrated consistent cardioprotective effects, ranging from the improvement of ventricular function in stable CHD to the mitigation of myocardial injury in acute settings, such as STEMI and post‐CABG surgery. Notably, the studies suggest that quercetin acts through distinct but complementary pathways: reducing systemic inflammation, limiting oxidative stress, and potentially addressing cellular senescence in the vascular endothelium. These findings collectively suggest that quercetin supplementation could serve as a valuable adjunct therapy to reduce both the physiological damage and the inflammatory sequelae associated with myocardial infarction and surgical intervention. The specific effects of quercetin on ischemia/reperfusion injury are summarized in Table 2 and illustrated in Figure 3.

**TABLE 2:** Effects of quercetin on cardiac ischemia/reperfusion.

Study design	Doses	Rout of administration	Effects	References
Cardiomyocyte	25, 50, and 100 mg/kg	Expose	↓Apoptosis ↑Silent information regulatory factor 1 (SIRT1) ↑Peroxisome proliferators‐activated receptor‐γ coactivator‐1α (PGC‐1α) ↑Bcl‐2 proteins expression ↓Bax protein expression	Tang et al. (2019)
Cardiomyocyte	Half‐maximal inhibitory concentration of 4.67 × 10–4 M	Expose	↑Ca2+ channels ↑Cellular mechanisms based on the L‐type Ca2+ channel (LTCC) ↑Myocardial contractility ↓Reactive oxygen species ↓Serum levels of myocardial enzymes ↓Catalase, glutathione, glutathione peroxidase ↓Amplitude of the cell shortening and Ca2+ transients ↑Cardioprotective effect ↓Ca2+ influx	Liang et al. (2020)
Cardiomyocyte	1 mM	Expose	↓Activator of transcription 3 (STAT3) ↓Caspase 9 ↓Intracellular reactive oxygen species production ↓Inflammatory factor and inducible MnSOD expression ↓Lipid metabolism. ↓Protein metabolism	Chen et al. (2013)
Rats	10 μM, 20 μM, 40 μM	Gavage	↑JAK2/STAT3 pathway ↓Cell apoptosis ↓Oxidative stress ↓Myocardial infarction ↑Ventricular remodeling	Liu, Yao, et al. (2021)
Rats	20 mg/kg/day		↓Systolic blood pressure ↑Vascular relaxatio ↑Expression of eNOS, PKCε expression	Ferenczyova et al. (2020)
Rabbits	1 mg/kg	Intravenous injection	↓NOX2, eNOS, and iNOS mRNA and protein expression ↓Myocardial ischemia–reperfusion‐↓ENOS mRNA and protein expression.	Wan et al. (2009)
Rats	1.0 mg/kg	Intravenous	↓Infarct volume ↓TNF‐alpha (TNF‐α) ↓Interleukin‐10 (IL‐10) ↓Inflammatory ↓MI/R injury primarily ↑Anti‐inflammatory effects	Jin et al. (2012)
Rats	100 nM	Intravenous	↑Left ventricular developed pressure (LVDP) ↓IL‐1β ↓TNF‐α ↓IL‐6 ↑Cardioprotective effects ↓Inflammatory reactions	Liu, Song, et al. (2021)
Rats	250 mg/kg	Oral	↓Apoptosis rate ↑Cardiac function ↓Aspartate aminotransferase (AST) ↓Lactate dehydrogenase (LDH) ↓Oxidative stress ↓Malondialdehyde (MDA) ↓Superoxide dismutase (SOD) ↓Catalase (CAT) ↓Inflammatory cytokines ↑Bcl‐2	Liu et al. (2014)
Rats	1 mg/kg	Add to buffer(reperfusion)	↓Inflammatory cytokine production ↓Myocardial injury ↑Heart function ↑Myocardial contractility	Dong et al. (2018)
Rats	20 mg/kg	Subcutaneously	↑Myeloperoxidase (MPO) activities ↓Glutathione (GSH) levels ↓SOD activities ↑Pro‐apoptotic marker caspase‐3 ↓Anti‐apoptotic bcl‐2 protein ↑Contractile responses to carbachol ↓Apoptosis induced by I/R.	Tinay et al. (2017)
Rats	10 mg/kg	Intraperitoneal	↓Infarct size ↓Lactate dehydrogenase ↓Caspase‐3 immunoreactivity ↑Akt phosphorylation ↑Bcl‐2 expression ↓Bax expression ↑Activating the PI3K/Akt signaling	Wang et al. (2013)
Mice	250 mg/kg/day	Gavage	↑Cardiac function ↓Myocardial injury ↓Infarct size ↓Myocardium oxidative damage ↓Apoptosis ↓IRI‐induced heart damage ↓NF‐κB pathway	Liu et al. (2016)
Rats	15 μM/L	Reperfusion	↑Postischemic recovery ↑Left ventricular developed pressure (LVDP) ↑End‐diastolic pressure	Monika Bartekova et al. (2010)
Mice	100 mg/kg/day	Gavage	↑Capillary density in the ischemic muscle ↑Angiogenesis in ischemic tissue	Sumi et al. (2013)
Rats	20 mg/kg/day	Investigation	↑Post‐ischemic recovery of LVDP ↑Markers of contraction and relaxation ↑+ (dP/dt)max and ‐(dP/dt)max	Bartekova et al. (2016)
Randomized clinical trial	120 mg/day	Oral supplement	↓ST segment depression ↓PVC number	Chekalina et al. (2017)
Randomized clinical trial	500 mg	Intravenous infusion	↓Creatine kinase‐myocardial band area ↓Reperfusion‐induced intramyocardial hemorrhage	Kozhukhov et al. (2024)
Randomized clinical trial	500 mg	Oral supplement	↓Vigorous inflammatory response Improve endothelial function	Dagher et al. (2021)
Randomized clinical trial	500 mg	Oral supplement	↓TNF‐α levels ↑Serum total antioxidant capacity Improved the insecurity dimension of the quality of life	Dehghani et al. (2021)

**FIGURE 3:**
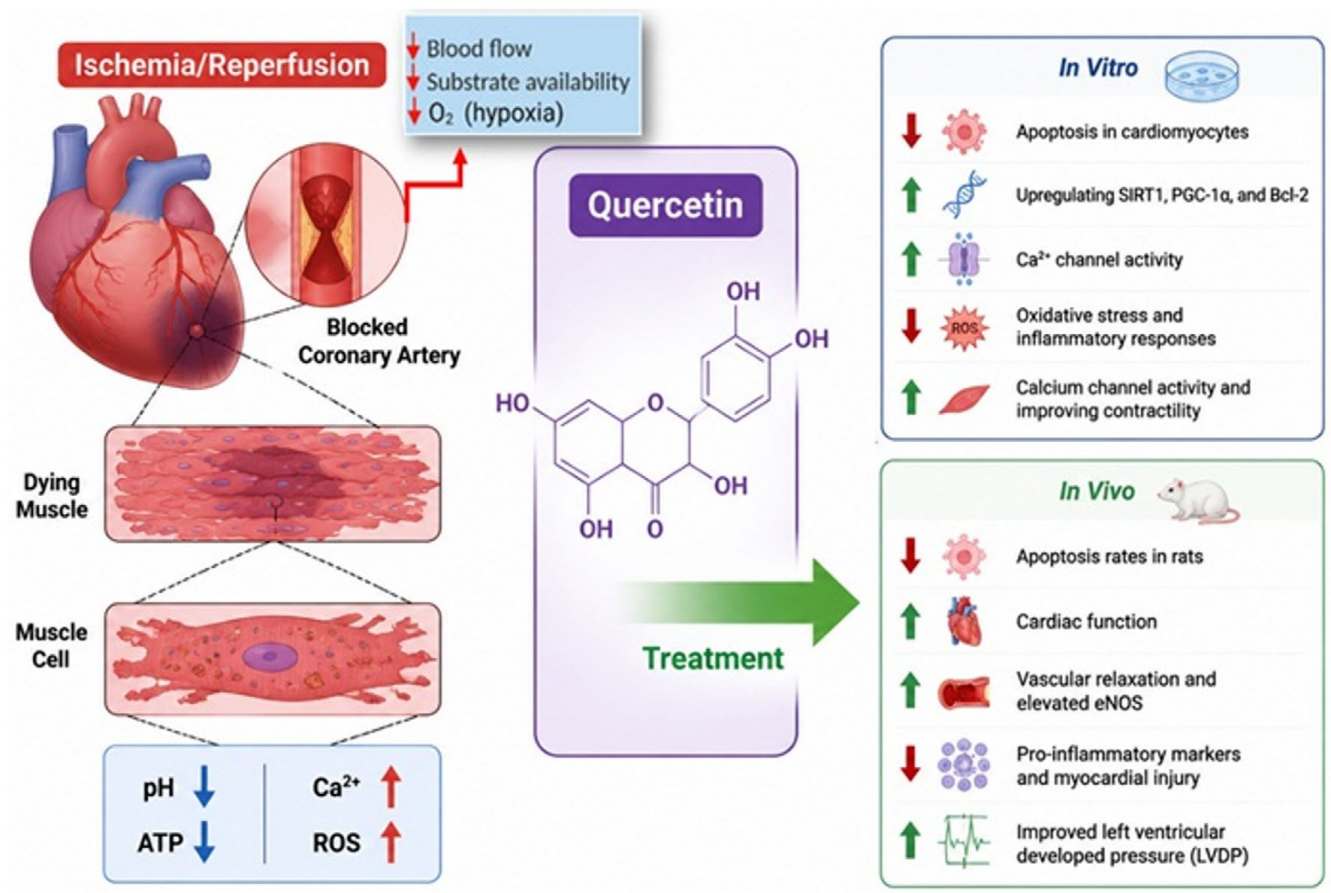
Effects of quercetin on ischemia/reperfusion.

## 4 Effects of Quercetin on Hypertension

### 4.1 In Vitro Studies

The effects of quercetin on pulmonary arterial hypertension (PAH) have been shown to significantly inhibit the proliferation of pulmonary artery smooth muscle cells (PASMC) induced by hypoxia, arrest cells at the G1/G0 phase, and reduce cell migration in a dose‐dependent manner. These findings indicate that quercetin enhances the levels of cyclin D1 protein while concurrently decreasing the expression of cyclin B1 and Cdc2. Furthermore, quercetin modifies the Bax/Bcl‐2 ratio and downregulates the expression of MMP2, MMP9, CXCR4, integrin β1, and integrin α5. Additionally, quercetin inhibits the TrkA/AKT signaling pathway, leading to reduced PASMC migration, cell cycle arrest, and the induction of apoptosis (He et al. 2015). In another study, quercetin demonstrated its ability to inhibit NADPH oxidase activity and reduce superoxide generation in vascular smooth muscle cells (VSMCs) derived from spontaneously hypertensive rats when administered at doses ranging from 1 to 100 μM (Jimenez et al. 2015). In mesenteric smooth muscle cells (MesSMC) from spontaneously hypertensive rats, treatment with a low concentration of quercetin (15 μmol/L) was found to mitigate AngII‐induced reactive oxygen species (ROS) production. Quercetin also diminished AngII‐stimulated NADPH oxidase activation and p47phox translocation to the cell membrane, significantly inhibiting both cell migration and proliferation (Redondo et al. 2012).

The effects of quercetin on pathological cardiac hypertension in rat cells model showed administration of 15 μmol/L by incubation significantly decreased AngII‐increased ROS production, AngII‐stimulated NAD(P)H oxidase activation, P47phox translocation to the cell membrane, co‐incubation of Q + C, migration and proliferation, and P38MAPK phosphorylation.

### 4.2 In Vivo Studies

In a rat model of pathological cardiac hypertension demonstrated that administering quercetin (10 mg/kg/day) via gavage significantly increased total non‐enzymatic antioxidant levels and SOD1 expression while reducing endothelial nitric oxide synthase (eNOS) expression (Castrejón‐Téllez et al. 2020). A higher dose of quercetin (30 mg/kg/day) over 3 weeks resulted in reduced inflammation and arteriole thickness. This treatment also significantly increased miR‐204 expression while reducing the levels of Poly (ADP‐ribose) polymerase 1 (PARP1), hypoxia‐inducible factor 1‐alpha (HIF1α), and nuclear factor of activated T cells c2 (NFATc2) proteins. All of which were elevated in the lung tissues of the control group suffering from pulmonary artery hypertension (Rajabi et al. 2020). Further analysis showed that administration of quercetin at doses of 10, 20, and 50 mg/kg/day resulted in decreases in both systolic blood pressure (SBP) and diastolic blood pressure (DBP), alongside reductions in fetal resorptions. Quercetin also contributed to increased fetal body weight and vascular endothelial growth factor (VEGF) levels. Furthermore, quercetin diminishing plasma concentrations of endothelin‐1 (ET‐1) and soluble fms‐like tyrosine kinase‐1 (sFlt‐1) in addition to lowering mRNA and protein levels of ET‐1 and endothelin‐1 type A receptor (ETAR) (Sun et al. 2020). Oral administration of quercetin (10 mg/kg/day) significantly decreased the number of vascular smooth muscle cells and the cross‐sectional area of the aortas. Similarly, quercetin treatment led to increased gelatinase activity and decreased ROS levels in the aortas of two‐kidney, one‐clip (2K1C) hypertensive rats (Pereira et al. 2018). Additionally, a combination treatment of quercetin and vitamin C in a rat model of cardiac hypertension improved the antioxidant defense system, decreased systolic and diastolic blood pressure, and reduced NF‐kB expression (Ajibade et al. 2017).

Quercetin administration (12.99 μg/mL) resulted in significant reductions in SBP, left ventricular and kidney weights, and serum levels of ACE activity, angiotensin‐II (ANG‐II), TNF‐α, NO, and IL‐6 in spontaneously hypertensive rats (Luo et al. 2017). Three‐week treatment of hypertensive rats with quercetin (30 mg/kg) significantly elevated miR‐204 expression, total antioxidant capacity, and Bcl‐2 protein levels while decreasing TNFα, IL‐6, malondialdehyde (MDA), and apoptosis markers (Rajabi et al. 2021).

The effects of the pathological cardiac model showed Oral administration of quercetin (10 mg/kg/day) for 3 weeks in hypertensive mice significantly increased antioxidant properties, NO, and NO formation. Also, it significantly reduced 2K1C‐hypertension‐induced increases in NADPH oxidase activity and vascular superoxide production (Montenegro et al. 2010). Quercetin at oral administration (10 mg/kg/day) for 10 in a rat model of pulmonary arterial hypertension days remarkably reduced mortality rates, pulmonary arterial pressure, and right ventricular hypertrophy (Morales‐Cano et al. 2014). Treatment of cardiac hypertension rats with quercetin (100 mg/kg/day) by subcutaneous injection significantly increased endothelial cell function and decreased mean pulmonary artery pressure (mPAP), right ventricular hypertrophy, and proliferating cell nuclear antigen (PCNA) expression (Gao et al. 2012).

The effect of quercetin (50, 100, and 150 mg/kg) for 2 weeks on NaCl‐induced hypertension in rats significantly reduced systolic, diastolic, pulse pressure, and heart rate in a dose‐dependent manner. The level of MDA was reduced, while the activities of the catalase, SOD, and GPx were increased in the treated hypertensive group (Olaleye et al. 2014). These results indicated that quercetin ameliorated hemodynamic and metabolic abnormalities induced by feeding rats a high‐salt diet.

### 4.3 Clinical Studies

Clinical studies have consistently demonstrated that oral quercetin supplementation exerts beneficial cardiovascular effects in hypertensive and pre‐hypertensive populations by improving endothelial function and attenuating systemic inflammation. Dower et al. reported that a dosage of 160 mg/day significantly improved endothelial health (Dower et al. 2015), while Brüll et al. observed that a 6‐week intervention (162 mg/day, derived from onion‐skin extract) significantly reduced 24‐h systolic blood pressure (BP), effective during both diurnal and nocturnal periods (Brüll et al. 2015).

In a study of 84 male patients with comorbid gout and essential hypertension, a 12‐month quercetin regimen demonstrated sustained antihypertensive efficacy, characterized by significant reductions in systolic BP, diastolic BP, and left ventricular mass index, alongside improvements in cardiac diastolic function and renal markers (Kondratiuk and Synytsia 2018).

Furthermore, Brüll et al. reported that 54 mg/day of quercetin enhanced antioxidant capacity, serum lipid profiles (triglycerides, total cholesterol), and the reactive hyperemia index (RHI) in hypertensive patients, while simultaneously decreasing markers of vascular endothelial activation, including endothelin‐1, intercellular adhesion molecule‐1 (ICAM‐1), and vascular cell adhesion molecule‐1 (VCAM‐1) (Brüll et al. 2017). Recent randomized controlled trials (RCTs) have further elucidated these effects. Moonikh et al. investigated the impact of quercetin (250 mg/day) for 2 months in men with hypertension following percutaneous coronary intervention (PCI). The intervention group showed significant improvements in oxidative stress parameters, BP regulation, and cardiac function compared to the placebo group (Moonikh et al. 2020). In an RCT involving adults aged ≥ 60 years, a 3‐month regimen of quercetin (240 mg/day), divided into three doses, significantly reduced body weight, body mass index (BMI), and both systolic and diastolic BP relative to baseline (Shatylo et al. 2021). Mantadaki et al. also evaluated the effects of daily quercetin supplementation (500 mg) for 12 weeks, followed by an 8‐week washout period in patients with type 2 diabetes mellitus. Findings revealed significant reductions in glycated hemoglobin (HbA1c) levels, systolic BP, and anxiety scores, alongside a measurable improvement in patient‐reported quality of life compared to controls (Mantadaki et al. 2024). Generally, quercetin appears to offer a multifaceted therapeutic approach to cardiovascular health by addressing both the symptomatic (blood pressure) and underlying (endothelial activation, oxidative stress, and insulin sensitivity) drivers of cardiovascular disease. While these findings are robust, the heterogeneity in dosage (ranging from 54 mg to 500 mg/day) and study duration underscores the need for future standardized clinical trials to establish optimal therapeutic protocols for specific patient cohorts. The effects of quercetin on hypertension are summarized in Table 3.

**TABLE 3:** Effects of quercetin on hypertension.

Study design	Doses	Rout of administration	Effects	References
Pulmonary artery smooth muscle cells (PASMCs) obtained from Sprague–Dawley rats	100 mg/kg	Oral	↓PASMC proliferation ↓Hypoxia‐induced PASMC proliferation ↓Cell migration ↑Cyclin D1 protein levels ↓Protein expression of cyclin B1 and Cdc2 ↓MMP2, MMP9, CXCR4, integrin β1, and integrin α5 expression ↓Activation of the TrkA/AKT signaling cascade	He et al. (2015)
Vascular smooth muscle cells (VSMCs) from spontaneously hypertensive rats	1–100 μM	Exposed	↑Antioxidant effects ↓Vascular superoxide ↑Endothelial function ↓O2(−) generation	Jimenez et al. (2015)
Mesenteric smooth muscle cells (MesSMC)	15 μmol/L	Incubation	↓AngII‐increased ROS production ↓AngII‐stimulated NAD(P)H oxidase activation ↓P47phox translocation to the cell membrane ↓Migration and proliferation	Redondo et al. (2012)
Rats	10 mg/kg/day	Oral	↑Insulin sensitivity ↓ENOS expression ↑Total non‐enzymatic antioxidant ↑SOD1 expression	Castrejón‐Téllez et al. (2020)
Rats	30 mg/kg/day	—	↓Arteriole thickness ↓Expression of PARP1, HIF1α, and NFATc2 proteins	Rajabi et al. (2020)
Rats	10, 20, and 50 mg/kg/day	Gavage	↓Inflammation ↓Platelet aggregation ↓Capillary permeability ↓Lipid peroxidation ↓SBP and DBP ↓ET‐1 and ETAR levels ↑VEGF expression ↓RUPP induced hypertension	Sun et al. (2020)
Rats	10 mg/kg/day	Gavage	↓Number of vascular smooth muscle cells aortas ↓ROS levels in aortas of 2K1C rats ↓Hypertension‐induced vascular remodeling ↓Oxidative stress ↓MMP‐2 activity in aortas	Pereira et al. (2018)
Rats	—	—	↑Antioxidants ↓SBP and DBP ↓NF‐kB expression	Ajibade et al. (2017)
Rats	12.99 μg/ml	Oral	↓SBP ↓Left ventricular ↓Kidney weight ↓Serum ACE activity ↓Angiotensin‐II (ANG‐II) ↓TNF‐α and IL‐6 ↓NO ↓ACE activity ↓Hypertension	Luo et al. (2017)
Rats	30 mg/kg	Diet	↑MiR‐204 expression ↑Total antioxidant capacity ↑Antiapoptotic protein Bcl‐2 ↓TNF‐α and IL‐6 ↓MDA	Rajabi et al. (2021)
Mice	10 mg/kg/day	Gavage	↑Antioxidants properties ↑Nitric oxide (NO) ↓2K1C‐hypertension ↑NO formation ↑Endothelial function	Montenegro et al. (2010)
Rats	10 mg/kg/day	Oral	↑Vasodilator ↑Antiproliferative effects ↓Oxidative status ↓Mortality ↓Arterial pressure ↓Right ventricular hypertrophy ↑Pulmonary artery vasodilation effects	Morales‐Cano et al. (2014)
Rats	100 mg/kg/day	Subcutaneous injection	↑Endothelial cell function ↓Mean pulmonary artery pressure (mPAP) ↓Right ventricular hypertrophy	Gao et al. (2012)
Rats	50, 100, and 150 mg/kg	Oral	↑Catalase, SOD, and GPx ↑Antihypertensive property ↓systolic, diastolic, pulse pressure ↓Heart rate ↓MDA	Olaleye et al. (2014)
Randomized clinical trial	160 mg/day	Oral supplement	↓Endothelial dysfunction ↓Inflammation ↑Improving endothelial function	Dower et al. (2015)
Randomized clinical trial	162 mg/day	Oral supplement	↓24‐h systolic BP ↓Daytime and nighttime systolic BP	Brüll et al. (2015)
Case‐ control	—	Oral supplement	↑Cardiac diastolic function ↑Antihypertensive effect ↓SBP, DBP, and LV mass index ↓Cardiac hypertension	Kondratiuk and Synytsia (2018)
Randomized clinical trial	54 mg	Oral supplement	↓Serum endothelin‐1 ↓Intercellular adhesion molecule‐1 ↑Serum triglycerides ↑Total cholesterol ↓HDL cholesterol ↑Plasma α‐tocopherol ↑Antioxidant capacity	Brüll et al. (2017)
Randomized clinical trial	250 mg/day	Oral supplement	↑Antioxidant capacity ↓Serum level of triglycerides ↓Serum level of total cholesterol	Moonikh et al. (2020)
Randomized clinical trial	240 mg/day	Oral supplement	↓Reduced body weight ↓Body mass index (BMI) ↓Systolic and diastolic BP	Shatylo et al. (2021)
Randomized clinical trial	500 mg	Oral supplement	↓Glycated hemoglobin (hba1c) levels ↓Systolic BP ↑Quality of life ↓Anxiety scores	Mantadaki et al. (2024)

## 5 Effects of Quercetin on Lipid Profile

### 5.1 In Vivo Studies

In a rat model subjected to high‐dose glucocorticoid treatment, administration of quercetin at doses of 50 and 150 mg/kg via gavage three times a week significantly improved total cholesterol (TC), triglyceride (TG), low‐density lipoprotein (LDL) levels, and the Apo B/A1 ratio. Moreover, quercetin administration reduced the TC/HDL, TG/HDL, and LDL/HDL ratios (Derakhshanian et al. 2020). When administered at a concentration of 0.33% (w/w) for 12 weeks in mice, quercetin was found to increase total polyunsaturated fatty acids (PUFA) and hepatic lipid metabolism while enhancing ω‐oxidation and decreasing circulating lipid levels. Quercetin also upregulated cytochrome P450 and acyl‐CoA thioesterase 3 (Hoek‐van den Hil et al. 2013).

Quercetin administration at a dietary dose of 185 mg/kg significantly decreased total body fat, liver weights, serum triglycerides, lipid accumulation, adipocyte size, and serum thiobarbituric acid levels, while increasing serum Trolox equivalent antioxidant levels in rats (Ting et al. 2018). In a rabbit model, dietary supplementation with 2 g of quercetin over 5–12 weeks significantly increased stearic acid (C18:0) and arachidonic acid (C20:4n‐6) levels (North et al. 2019). Merino lambs receiving quercetin (185 mg/kg) exhibited a significant increase in rumenic acid content (9c,11t‐18:2), alongside reductions in saturated fatty acids and lipid peroxidation in meat (Andrés et al. 2014). Furthermore, administration of quercetin (60 mg/kg, intraperitoneally) effectively reduced lipid hydroperoxides and enhanced the activity of antioxidant enzymes, including glutathione peroxidase, in a rat model (Pereira Braga et al. 2013).

The combination of dietary quercetin at doses of 100, 200, and 300 mg/kg with α‐tocopherol significantly enhanced growth performance, antioxidant capacity, and lipid stability in male broiler birds, improving the overall fatty acid composition, particularly saturated fatty acids (Sohaib et al. 2015). Supplementation with 0.33% (w/w) quercetin for 12 weeks resulted in decreased hepatic lipid accumulation, serum lipid levels, and weight gain, along with reduced expression of cytochrome P450 2b (Cyp2b) genes, which are regulated by the transcription factor constitutive androstane receptor in male mice (Hoek‐van den Hil et al. 2014).

Oral administration of quercetin at a dose of 10 mg/kg for 30 days led to increases in serum HDL and tissue phospholipid levels, while significantly lowering serum cholesterol, triglycerides, LDL, very low‐density lipoprotein (VLDL), and tissue triglyceride and cholesterol levels in rats (Padma et al. 2012). These studies involve different models that provide foundational evidence for the conserved metabolic mechanisms by which quercetin modulates lipid metabolism. Specifically, these studies collectively demonstrate that quercetin consistently improves lipid profiles, decreasing atherogenic saturated fatty acids and increasing beneficial polyunsaturated fatty acids. The shift in fatty acid composition observed in these studies (e.g., reduction in saturated fats and lipid peroxidation) by quercetin is a well‐established surrogate for improved cardiovascular outcomes.

### 5.2 Clinical Studies

In a randomized clinical trial involving 60 male subjects over 8 weeks, the combination of quercetin and vitamin C (500 mg/day and 200 mg/day, respectively) resulted in significant decreases in LDL cholesterol levels (Askari et al. 2013). Administration of quercetin on cardiovascular risk factors and inflammatory biomarkers in women with type 2 diabetes was carried out in a RCT for 10 weeks. Treatment with quercetin capsule (500 mg) daily decreased systolic blood pressure significantly. High‐density lipoprotein cholesterol (HDL‐C) was significantly decreased in both groups. Also, quercetin supplementation significantly reduced the serum concentration of TNF α and IL‐6 compared to the control group (Zahedi et al. 2012).

Nishimura et al. evaluated the effect of daily intake of quercetin‐rich onion powder on visceral adiposity over a 12‐week period. In this RCT involving 70 healthy subjects (BMI ≥ 23 and < 30 kg/m^2^), participants ingested 9 g of onion powder daily. While no significant difference in total visceral fat area (VFA) was observed between the treatment and placebo groups, a sub‐group analysis revealed that participants with lower baseline high‐density lipoprotein (HDL) cholesterol levels exhibited a significant reduction in VFA following the quercetin‐rich onion intervention (Nishimura et al. 2019).

The potential independent and additive effects of α‐linolenic acid (ALA) and quercetin on blood pressure, lipid and glucose metabolism, and biomarkers of inflammation were investigated. In an RCT crossover trial, healthy volunteers received either 3.6 g/day of ALA +190 mg/day of quercetin or a placebo for 8 weeks. The combination intervention significantly decreased total cholesterol, low‐density lipoprotein (LDL) cholesterol, non‐HDL cholesterol, and apolipoprotein B concentrations compared to the placebo group (Burak et al. 2019). A meta‐regression analysis of five randomized controlled trials (RCTs) assessing the effects of quercetin on plasma lipids indicated that supplementation at doses ≥ 500 mg/day for at least 4 weeks significantly reduced total cholesterol (*p* = 0.04) and triglycerides (*p* < 0.0001), while LDL‐C and HDL‐C concentrations remained unchanged (*p* > 0.05). Notably, changes in plasma triglyceride levels were significantly associated with both dosage (*p* = 0.02) and the duration of quercetin supplementation (*p* = 0.01) (Sahebkar 2017). The clinical data presented herein demonstrate that quercetin supplementation exerts a favorable influence on lipid metabolism and systemic inflammation, particularly when administered at doses ≥ 500 mg/day. These findings highlight quercetin's potential as a therapeutic adjunct in managing cardiovascular risk, with effects that appear both dose‐ and duration‐dependent. Quercetin acts as a multifaceted pharmacological candidate capable of ameliorating both lipid metabolism and inflammatory cascades. The synergistic effects observed when combining quercetin with other nutraceuticals, such as vitamin C or α‐linolenic acid, suggest that multi‐nutrient strategies may optimize therapeutic outcomes. The effects of quercetin on lipid profiles are summarized in Table 4.

**TABLE 4:** Effects of quercetin on lipid profile.

Study design	Doses	Rout of administration	Effects	References
Rats	150 mg/kg	Gavage	↓Apo B/A1 ratio ↓TC/HDL ↓TG/HL ↓LDL/HDL	Derakhshanian et al. (2020)
Mice	0.33% (w/w)	Diet	↓Triglycerides (TG) ↑Total poly unsaturated fatty acids (PUFA) ↓Palmitic acid ↑Hepatic lipid metabolism ↑ω‐oxidation ↑Cytochrome P450 oxidoreductase	Hoek‐van den Hil et al. (2013)
Rats	185 mg/kg	Feed	↓Total body fat ↓Liver weights ↓Serum TG ↓Lipid accumulation ↓Serum thiobarbituric acid ↑Serum Trolox equivalent antioxidant	Ting et al. (2018)
Rabbits	2 g dihydrate/kg	Feed	↑C18:0 in the fat ↑C20:4n‐6	North et al. (2019)
Merino lambs	185 mg/kg	Feed	↑Rumenic acid content (9c,11 t‐18:2) ↓Saturated FA ↓Lipid peroxidation	Andrés et al. (2014)
Rats	60 mg/kg	Intraperitoneal injection	↓Lipid hydroperoxides ↑Activity of the antioxidant enzyme glutathione peroxidase	Pereira Braga et al. (2013)
Male broiler birds	100, 200, 300 mg/kg	Feed	↓Fatty acid generation ↑Antioxidant capacity, stability of lipids and fatty acid composition	Sohaib et al. (2015)
Adult male mice	0.33% (w/w)	Diet	↓Serum lipid levels ↓Cytochrome P450 2b (Cyp2b) genes ↓High‐fat diet ↓Serum lipid levels	Hoek‐van den Hil et al. (2014)
Wistar rats	10 mg/kg	Orally	↓Low density lipoprotein (LDL) ↓Very Low Density Lipoprotein (VLDL) ↓Tissue triglycerides ↓Cholesterol ↑Serum HDL ↑Tissue phospholipids	Padma et al. (2012)
Humans	500 mg/day	Diet	↓Low density lipoprotein (LDL)	Askari et al. (2013)
Humans	≥ 500 mg/day	Diet	↓Total cholesterol ↓Triglycerides	Sahebkar (2017)

### 5.3 Limitations

Several limitations inherent in the current body of literature must be acknowledged. First, the significant methodological heterogeneity in experimental studies regarding administration routes (e.g., oral gavage vs. subcutaneous or intraperitoneal injection), treatment durations (ranging from 10 days to 4 weeks) complicates the establishment of a standardized therapeutic protocol. Second, the pharmacokinetic profile of quercetin—specifically it's notoriously low and variable oral bioavailability—introduces significant uncertainty when comparing dose–response outcomes across different studies. Third, the pathophysiological diversity of the different animal models used means that the observed molecular changes may reflect model‐specific compensatory mechanisms rather than a common pharmacological effect.

## 6 Conclusion

Quercetin as a natural flavonoid exhibits substantial therapeutic potential against hypertrophy, ischemia/reperfusion injury, and hypertension through its multifaceted modulation of oxidative, inflammatory, and apoptotic pathways in both in vitro and in vivo studies. While initial clinical trials demonstrate its potential to improve lipid profiles and left ventricular function, the current body of literature remains heterogeneous, partly due to variability in study design and patient populations. The primary translational barrier to quercetin's clinical utility remains its poor aqueous solubility, rapid metabolism, and low systemic bioavailability. Future research must base toward overcoming these pharmacological constraints. Specifically, the development of advanced delivery systems such as lipid‐based nanoparticles, liposomes, and nano‐emulsions represents a critical frontier to enhance the bioavailability and targeted delivery of quercetin to cardiac tissue. Consequently, quercetin is currently viewed as a potential complementary agent in cardiovascular risk management; however, large‐scale, long‐term studies are essential to elucidate its therapeutic efficacy, safety profile, and underlying mechanisms of action across diverse populations.

## Author Contributions

**Mohammad Reza Khazdair:** conceptualization, writing – original draft, validation, writing – review and editing, supervision. **Shima Jafari:** conceptualization, writing – original draft, data curation, visualization. **Melika Shoghi:** conceptualization, writing – original draft, data curation.

## Funding

The authors have nothing to report.

## Consent

The authors have nothing to report.

## Conflicts of Interest

The authors declare no conflicts of interest.

## Data Availability

Data sharing is not applicable to this article, as no new data were created or analyzed in this study.
